# Tolerance by Surprise: Evidence for a Generalized Reduction in Prejudice and Increased Egalitarianism through Novel Category Combination

**DOI:** 10.1371/journal.pone.0057106

**Published:** 2013-03-06

**Authors:** Milica Vasiljevic, Richard J. Crisp

**Affiliations:** 1 Centre for the Study of Group Processes, School of Psychology, University of Kent, Canterbury, Kent, United Kingdom; 2 Department of Psychology, University of Sheffield, Sheffield, United Kingdom; University of Bologna, Italy

## Abstract

Prejudices towards different groups are interrelated, but research has yet to find a way to promote tolerance towards multiple outgroups. We devise, develop and implement a new cognitive intervention for achieving generalized tolerance based on scientific studies of social categorization. In five laboratory experiments and one field study the intervention led to a reduction of prejudice towards multiple outgroups (elderly, disabled, asylum seekers, HIV patients, gay men), and fostered generalized tolerance and egalitarian beliefs. Importantly, these effects persisted outside the laboratory in a context marked by a history of violent ethnic conflict, increasing trust and reconciliatory tendencies towards multiple ethnic groups in the Former Yugoslav Republic of Macedonia. We discuss the implications of these findings for intervention strategies focused on reducing conflict and promoting peaceful intergroup relations.

## Introduction

The spectre of prejudice can rapidly reverse the harmony of intergroup relations, and escalate into full-scale conflict, war and genocide. The Oxford English Dictionary (2011) defines prejudice as: *“a preconceived opinion that is not based on reason or actual experience”.* In this article we devise, develop, and test a new approach to reducing prejudice that targets this unreasoning, *heuristic* basis for prejudice. Social cognitive research has shown that the roots of prejudice are buried deep in a fundamental bias in the way people process information. The intervention we propose does not attempt to change the content of existing stereotypes, it changes *the way in which people think* about outgroups. We argue that this ‘core cognition’ approach to reducing prejudice has great potential because it addresses one of the biggest challenges facing contemporary research on prejudice-reduction: How to promote *generalized* tolerance towards multiple groups; that is, egalitarianism in intergroup attitudes.

### Achieving Generalized Tolerance

For decades, social scientists have been concerned with the question how to reduce prejudice between social groups. Much progress has been made, but the field faces an important challenge: Techniques that reduce prejudice towards one group do not readily transfer to other outgroups, or in other words, promote *generalized* tolerance. Consider research arising from Allport's [1] contact hypothesis: The prejudice-reducing effects of contact may be beneficial to the target outgroup (e.g., immigrants), but do not routinely generalize to other outgroups (e.g., the disabled). Although there is some recent evidence for so-called secondary transfer effects, this transfer has been limited to groups that share the same superordinate category (e.g., immigrants and political refugees) [2]. Our aim was to develop a new intervention designed specifically to foster *generalized* tolerance. In so doing, we hope to provide a new type of cognitive intervention to fill the gap between existing contact [3], [4], and multicultural [5], [6], [7], [8], perspectives on prejudice reduction. Our approach is rooted in scientific research on the categorical basis of person perception, so this is where our treatise begins.

### Multiple Social Categorization

Categorizing people into different groups, “us” and “them”, has been the basis of intractable conflict across the world. *The Troubles* between the Catholics and the Protestants in Northern Ireland, the long drawn Israeli-Palestinian conflict in the Middle East, and the ethnic cleansing between the Croats, Serbs, and Bosnian Muslims on the territory of the Former Yugoslavia are but a few examples. Research on multiple social categorization has explored what happens when instead of this simple “us” vs. “them” criterion, people are compelled to think in ways that emphasize *multiple* affiliations [9], [10]. Recent research on multiple categorization has shown that encouraging people to think of counter-stereotypic categorizations is particularly effective at reducing prejudice [11], [12]. Counter-stereotypic categorization describes when a person does not fit in to existing categorical expectancies (e.g., a gay priest, a male midwife). Consistent with underlying social cognitive theory [13], when a person is described by mutually (stereotypically) inconsistent categories, perceivers cognitively ‘shift gear’ to focus on individuating characteristics as a way of resolving the inconsistency [14], [15]. Our contention is that this cognitive switching from heuristic to individuated thinking does not stop with the target at hand, but has the potential to be a much more powerful approach to prejudice-reduction than previously thought. The hypothesized extended benefits of this heuristic switching, under counter-stereotypic conditions, are derived from research on the cognitive characteristics of *mindsets*.

### Mindsets and Intergroup Conflict

Mindsets are content-free processing orientations that are often linked to goals. For example, individuals can be motivated to integrate or differentiate information, leading to assimilation and contrast effects respectively [16]. Mindsets are also linked to different stages of goal-pursuit [17], [18], [19], and have important self-regulatory functions [20]. Mindsets impact judgments *independently* of the context in which they were elicited, and it is precisely for this reason that methods that tackle people’s core cognitions may be more successful at reducing prejudice in real contexts of conflict. One notable example is recent research carried out in the context of the Israeli-Palestinian conflict by Halperin and colleagues [21] which aimed to change people’s beliefs about outgroup malleability. In a cross-sectional field study of Israeli Jews they first showed that believing groups were malleable led to more positive attitudes, which in turn led to a greater willingness to compromise with the Palestinians. Across three further experiments Halperin and colleagues showed that inducing individuals with beliefs about the malleable versus fixed nature of groups encouraged more positive attitudes towards the outgroup, which then led to greater willingness to compromise for peace. Notably, these results emerged amongst diverse samples (i.e., Israeli Jews, Palestinian citizens of Israel, and Palestinians in the West Bank) attesting to the convergent validity of this technique to reduce prejudice via modifying people’s beliefs. That the intervention did not mention specific adversary groups during the induction stage, speaks to the viability of developing interventions that tackle people’s core cognition (i.e., the mindset they adopt when thinking about outgroups) rather than the content of their specific prejudices.

Speaking to the potential mechanisms through which mindsets may affect social perception, Sassenberg and Moskowitz [22] found that priming individuals with a creative mindset can inhibit the automatic activation of stereotypes at an implicit level. In their studies, participants who were primed to adopt a creative mindset showed lowered automatic activation of stereotypes associated with African Americans when compared to participants who were primed to adopt a thoughtful mindset (Study 1), and also showed decreased activation of stereotypes related to neutral non-social stimuli (Study 2). These findings illustrate that mindsets can promote changes to individuals’ core cognition when confronted with information on outgroup members.

### A Heuristic-Switching Mindset

We argue that the process of individuation, outlined by Fiske and Neuberg’s [13] continuum model, and evident under counter-stereotypic conditions, may be much more powerful than previously thought. If conceptualized, and harnessed, as a mindset manipulation, we argue that counter-stereotypes can elicit a heuristic-switching mindset, which will result in a temporary, cognitive shift away from heuristic thinking [23]. Such a mindset may be key to achieving *generalized* tolerance because adopting such a mindset will promote the temporary tendency to think of *all* groups not in heuristic, stereotypic terms, but as individuals. Specifically, we hypothesize that being compelled to think counter-stereotypically about others should induce a thinking style characterized by the tendency to abandon established routines (i.e., stereotyping), engage in generative thought, and consider individuating attributes, *regardless of the specific target group at hand*.

Our predictions derive from the Categorization-Processing-Adaptation-Generalization model (CPAG) [23], which proposes that experiencing diversity that confronts existing stereotypes promotes a shift from heuristic modes of thinking, thereby lessening people’s reliance on stereotypes in guiding evaluations of groups. According to the CPAG model such a heuristic-switching mindset will only ensue if people are motivated to engage with the stereotype-disconfirming information, and have the cognitive resources to resolve the inconsistency.

### The Present Research

One aim of the present investigation was to test the notion, derived from the CPAG model, that experiencing social diversity that challenges people’s preconceptions can promote generalized tolerance. In its focus on promoting cognitive flexibility this proposition links with Sassenberg and Moskowitz’s [22] earlier work; however, it goes much further to specify uniquely how cognitively flexible responding can (a) be manifested in increased tolerance evidenced across multiple outgroups and (b) can be encouraged through the experience of counter-stereotypic category combination. As such, our model provides a way of linking research on the cognitive underpinnings of tolerance to research on social categorization and social diversity, with corresponding implications for multicultural policy and practice.

An important goal of the present research was also to examine, for the first time, the consequences of resolving inconsistencies for a *generalized* reduction in prejudice and increased egalitarianism. This is important for at least two reasons. First, previous work on cognitive flexibility mindsets did neither examine social judgments (i.e., the *application* of stereotypes), nor the wider implications for promoting tolerance and egalitarian attitudes. Secondly, and perhaps most importantly, at the present there are no interventions to tackle prejudicial perceptions towards multiple outgroups that do not share the same superordinate category. This represents a significant gap in our knowledge of prejudice reduction, which the present research seeks to fill.

We devised a task that asked participants to generate either five counter-stereotypic, or five stereotypic, social category combinations (see Supporting Material S1 for a copy of the cognitive task), see also [24]. Participants were free to generate any social category combinations they could think of. Examples of counter-stereotypic combinations generated by participants include: *overweight model, rich student, female firefighter*, or *male midwife*. Generation of stereotypic combinations was the appropriate control because it constituted a task of equivalent load while representing the default mode of stereotypic person perception [13], [25], [26], [27]. We therefore hypothesized that generating five counter-stereotypic category combinations would elicit greater cognitive flexibility and engender generalized tolerance toward a range of outgroups. To then establish whether the benefits of a counter-stereotypic mindset extend beyond the laboratory, we also conducted a field experiment in a context marked by a history of ethnic conflict. All six experiments were conducted in accordance with APA standards for the ethical treatment of human participants, and gained the prior approval by the Ethics Committee of the School of Psychology at the University of Kent. Informed written consent was obtained from all participants involved in these experiments. The six experiments we conducted are reported below.

## Experiments 1, 2, and 3: Cognitive Foundations

The first three experiments aimed to develop the new procedure and test the underlying assumptions of our theoretical model. Specifically, Experiment 1 tested whether generating surprising category combinations activated generative thought and a cognitive flexibility mindset as evidenced by a lowered *need for cognitive closure* [28]. The need for cognitive closure reflects an individual’s preference for a concrete solution as opposed to enduring uncertainty and ambiguity. Need for closure is an important concept for the present purposes because individuals with a low need for closure are more inclined to deliberate and seek out novel information [29], focus more on individuating information as opposed to categorical information [30], and rely less on immediate impressions and stereotypic knowledge [31], [32].

Experiment 2 probed the consequences of this mindset for the inhibition of stereotypic (i.e., dominant) associations using the Stroop paradigm [33]. Performance on the Stroop test reflects multiple underlying capacities, but an important component is the ability to inhibit the processing of semantic content. The task also requires individuals to adjust to different task demands. Thus, the Stroop task was our preferred choice to provide an index of people’s capacity to exhibit cognitive control and to respond to the changing demands of their environment.

Experiment 3 employed a measure of *lateral thinking* [34] to confirm that a counter-stereotypic mindset encourages flexible, divergent thinking. Lateral thinking is the aptitude to use an indirect and inventive approach when faced with the task of solving problems. Thus, lateral thinking involves observing the problem at hand from multiple, novel perspectives, discarding traditional modes of thinking. Unlike Experiments 1 and 2, Experiment 3 also employed a baseline condition in which participants did not generate any social category combinations.

### Method

#### Participants and design

In Experiment 1, fifty British undergraduates (22 females, *M_age_*  = 20.96), in Experiment 2, sixty-one British undergraduates (47 females, *M_age_* = 18.97), and in Experiment 3, fifty-four British undergraduates (43 females, *M_age_* = 20.53) were randomly assigned to a counter-stereotypic or stereotypic priming condition. In Experiment 3, we added a second control condition in which participants did not generate any category combinations. Unless stated otherwise, course credits were offered in return for participation in all experiments.

#### Procedure and materials

Upon arrival participants were asked to write down five counter-stereotypic, or five stereotypic social category combinations. In Experiment 3, a third group of participants did not complete this step. In all experiments, manipulation checks confirmed that participants primed with counter-stereotypicality rated the category combinations they generated as more surprising and less similar than the participants primed with stereotypicality (all *p*s<.01). In Experiment 1, participants then completed the *Need for Cognitive Closure – Lexical* scale (*NFCC-L*) [28]. It required participants to choose one of two possible words to complete a sentence, i.e., “She preferred to travel to [familiar, unfamiliar] places” (coded: 0-*ambiguous*, 1-*concrete*) [*α* = .69, *M* = .50, *SD* = .18]. In Experiment 2, participants completed a standard computerized *Stroop* task after the priming task [33]. In Experiment 3, participants were asked to solve ten puzzles that required *lateral thinking* (e.g., Question: “A police officer saw a truck driver clearly going the wrong way down a one-way street, but did not try to stop him. Why not?”; Answer: Because the truck driver was walking). Answers were scored for accuracy on a dichotomous scale (0 = *incorrect*, 1 = *correct*). A composite score was derived by summing up all correct responses whilst taking into account participants’ prior familiarity with the puzzles.

### Results and Discussion

In Experiment 1, an independent samples *t*-test confirmed that participants primed with a counter-stereotypic mindset displayed a lower need for cognitive closure than those primed with stereotypicality, *t*(48) = 2.05, *p* = .046, d = 0.59 (*Ms* = .46 vs..56). These results provide a first indication that priming a counter-stereotypic mindset is conducive to generative thought and a move away from heuristic forms of thinking - a basis for greater cognitive flexibility [35]. Because a low need for cognitive closure is associated with lowered outgroup derogation [36], these findings also hint at the possibility that priming a counter-stereotypic mindset may lead to decreases in prejudice.

Standard pre-analysis treatment of response times in the Stroop task in Experiment 2 were performed, resulting in the removal of four outliers with response times exceeding two and a half standard deviations the sample average. An independent samples *t*-test then showed that generating counter-stereotypic category combinations reduced Stroop interference (*Ms* = 69 ms vs. 100 ms), *t*(55) = 1.89, *p* = .06, d = 0.51. This further indicates a switch away from heuristic thinking under counter-stereotypic conditions, and the enhanced tendency to engage executive functions such as inhibition of dominant associations (i.e., stereotypes).

In Experiment 3, a one-way ANOVA demonstrated that there were significant differences between the three experimental conditions, *F*(2, 53) = 4.50, *p* = .016, η^2^ = 0.15. Tukey post-hoc comparisons revealed that participants primed with counter-stereotypicality (*M* = 3.50) solved significantly more lateral thinking puzzles compared to participants primed with stereotypicality (*M* = 2.29), *p* = .038; and also compared to participants who did not generate any category combination prior to the puzzles (*M* = 2.26), *p* = .027. Comparisons between the baseline and stereotypic condition were not significant, *p* = .998. These results demonstrate that thinking of counter-stereotypic exemplars can lead to a more indirect and creative approach when solving problems. Moreover, this study further supports the contention that it is priming counter-stereotypic thinking, not stereotypic thinking, that leads to changes in peoples’ core cognitive style.

## Experiment 4: Reducing Prejudice and Promoting Tolerance

The main aim of Experiment 4 was to probe the consequences of a counter-stereotypic mindset for promoting tolerance and reducing prejudice towards *multiple* outgroups [37]. To determine the scope of the intervention for promoting tolerance, we assessed individuals’ commitment to democratic norms [38]. In addition, with the view to maximizing the impact of the priming procedure, we varied the number of category combinations participants were required to generate. On the one hand, generating more surprising category combinations allows for greater practice and longer exposure to counter-stereotypic thought, and this would argue for stronger effects with an increased number of category combinations. On the other hand, generating more category combinations implies greater effort, which could undermine the benefits of the priming task through cognitive depletion, or by reducing individuals’ confidence in their thought processes [39]. In light of these conflicting predictions, we asked participants to generate either five (easy) or ten (difficult) category combinations.

### Method

#### Participants and design

Eighty-three British undergraduates (53 females, *M_age_ = *23.49) were randomly assigned to the conditions of a 2 (combination type: counter-stereotypic vs. stereotypic)×2 (number of combinations: five vs. ten) factorial design.

#### Procedure and materials

After having generated five (ten) counter-stereotypic (stereotypic) category combinations, participants rated their attitudes towards different social outgroups: *elderly*, *disabled*, *HIV patients*, *asylum seekers*, and *gay men,* using the following bipolar adjective pairs separated by a 9-point scale: *warm-cold*, *negative-positive*, *friendly-hostile*, *suspicious-trusting*, *respect-contempt*, *admiration-disgust* (*General Evaluation Scale*) [37]. At the end, participants indicated their *Commitment to Democratic Norms* [38] (e.g., “Free speech should be provided for all no matter what their views might be”) using a seven-point scale (1 = *strongly disagree;* 7* = strongly agree*) [*α* = .71, *M* = 5.73, *SD* = .86].

### Results and Discussion

A 2 (combination type: counter-stereotypic vs. stereotypic)×2 (number of combinations: five vs. ten) MANOVA was computed on the evaluation ratings for the five minority outgroups after reverse coding of negative items. The analysis yielded a significant interaction between combination type and number of combinations, *F*(5, 74) = 2.34, *p* = .050, η^2^ = 0.05. Simple effects confirmed that, in the five combinations condition, thinking counter-stereotypically led to more favorable attitudes towards *all the outgroups* (*Ms* = 6.42 vs. 5.79), *F*(5, 74) = 2.37, *p* = .047, d = 0.34. In other words, thinking counter-stereotypically promoted generalized tolerance. No such effect was found in the ten combinations condition (*Ms* = 6.16 vs. 6.36), *F*<1. Turning back to the omnibus test, no other effects emerged, *Fs*<1.6 (see Table 1). A repeated measures ANOVA with combination type and number of combinations as between-subjects factors revealed that the effects of the surprising categories priming did not differ between the five target groups, *F*(4, 304) = 1.39, *p* = .235, *ε = *.90.

**Table 1:** Attitudes towards different outgroups as a function of combination type and number of combinations (Experiment 4).

	Category Combination			
	Stereotypic		Counter-Stereotypic	
Outgroup	*M*	*SD*	*M*	*SD*
	Five Combinations			
Elderly	6.64	1.34	7.11	1.43
Disabled	6.18	1.45	7.31	1.16
HIV patients	5.64	1.75	6.60	1.31
Asylum seekers	4.95	1.68	6.12	1.34
Gay men	6.55	1.52	6.79	1.71
	Ten Combinations			
Elderly	7.17	.81	6.84	1.07
Disabled	6.83	1.32	6.35	1.34
HIV patients	6.35	1.68	5.97	1.62
Asylum seekers	5.83	1.52	5.31	1.62
Gay men	6.33	1.93	6.33	1.73

A 2 (combination type: counter-stereotypic vs. stereotypic)×2 (number of combinations: five vs. ten) ANOVA was conducted on Commitment to Democratic Norms. The analysis yielded a significant interaction, *F*(1, 77) = 4.31, *p* = .041, η^2^ = 0.05. Analyses of simple effects revealed that after generating five counter-stereotypic category combinations participants were more committed to democratic norms than after generating five stereotypic category combinations (*Ms* = 6.06 vs. 5.44), *F*(1, 77) = 5.77, *p* = .019, d = 0.54. No such effect was found when participants generated ten combinations, (*Ms* = 5.63 vs. 5.79), *F*<1. No other significant effects emerged.

As predicted the results showed that priming participants with counter-stereotypic thinking led to more positive attitudes towards a diverse range of outgroups and, notably, increased individuals’ commitment to democratic norms. No such effect was found when participants generated ten category combinations. This suggests that the benefit of counter-stereotypic over stereotypic thought diminishes as individuals are compelled to generate a large number of category combinations. Thus, although with the current sample size generating a smaller number of counter-stereotypic exemplars did not yield a significant difference from generating many counter-stereotypic exemplars (*p = *.172), the overall pattern of results supports the notion that generating a lower versus higher number of combinations may be the optimal strategy to achieve a generalized reduction in prejudice.

## Experiment 5: Egalitarianism through Flexible Thought

Experiments 1–3 demonstrated that counter-stereotypic thinking improves lateral thinking, lowers the need for cognitive closure and helps overcome dominant associations. Experiment 4 provided the first evidence that these characteristics of the hypothesized mindset result in generalized tolerance across multiple outgroups. The aim of Experiment 5 was to demonstrate more directly that generating counter-stereotypic category combinations encourages individuals to embrace diversity and to do away with rigid preconceptions. To this end, we examined the moderating role of *Personal Need for Structure (PNS)* [40]. If generating counter-stereotypic category combinations increases tolerance by compelling perceivers to push stereotypic thinking aside, then individuals with a low need for structure should respond best to the experimental procedure and exhibit a larger increase in tolerance than individuals with a high need for structure, who may be reluctant to do away with stereotypic preconceptions. To test these predictions, we focused on another facet of tolerance: endorsement of egalitarian values. For this purpose we utilized Katz and Hass’s [41] *Humanitarianism-Egalitarianism* scale, which measures peoples’ endorsement of equality of opportunity, social justice, and concern for the well-being of other individuals regardless of their respective group membership. As in the previous experiment, we also asked participants to generate either five or ten category combinations.

### Method

#### Participants and design

Eighty British undergraduates (63 females, *M_age_ = *20.85) participated in the experiment. The design was identical to Experiment 4.

#### Procedure and materials

Upon arrival participants completed the *Personal Need for Structure* scale (*PNS*) [40] (e.g., “I don't like situations that are uncertain”; 1 = *strongly disagree* to 6 = *strongly agree*; *α* = .78, *M* = 3.71, *SD* = .61) prior to generating five (ten) counter-stereotypic (stereotypic) category combinations. Next, participants completed the *Humanitarian-Egalitarian scale* [41] (*α* = .87, *M* = 5.49, *SD* = .82), which measured participants’ egalitarian value orientations (e.g., “One should be kind to all people”) [1 = *strongly disagree,* 7 = *strongly agree*].

### Results and Discussion

**Figure 1:**
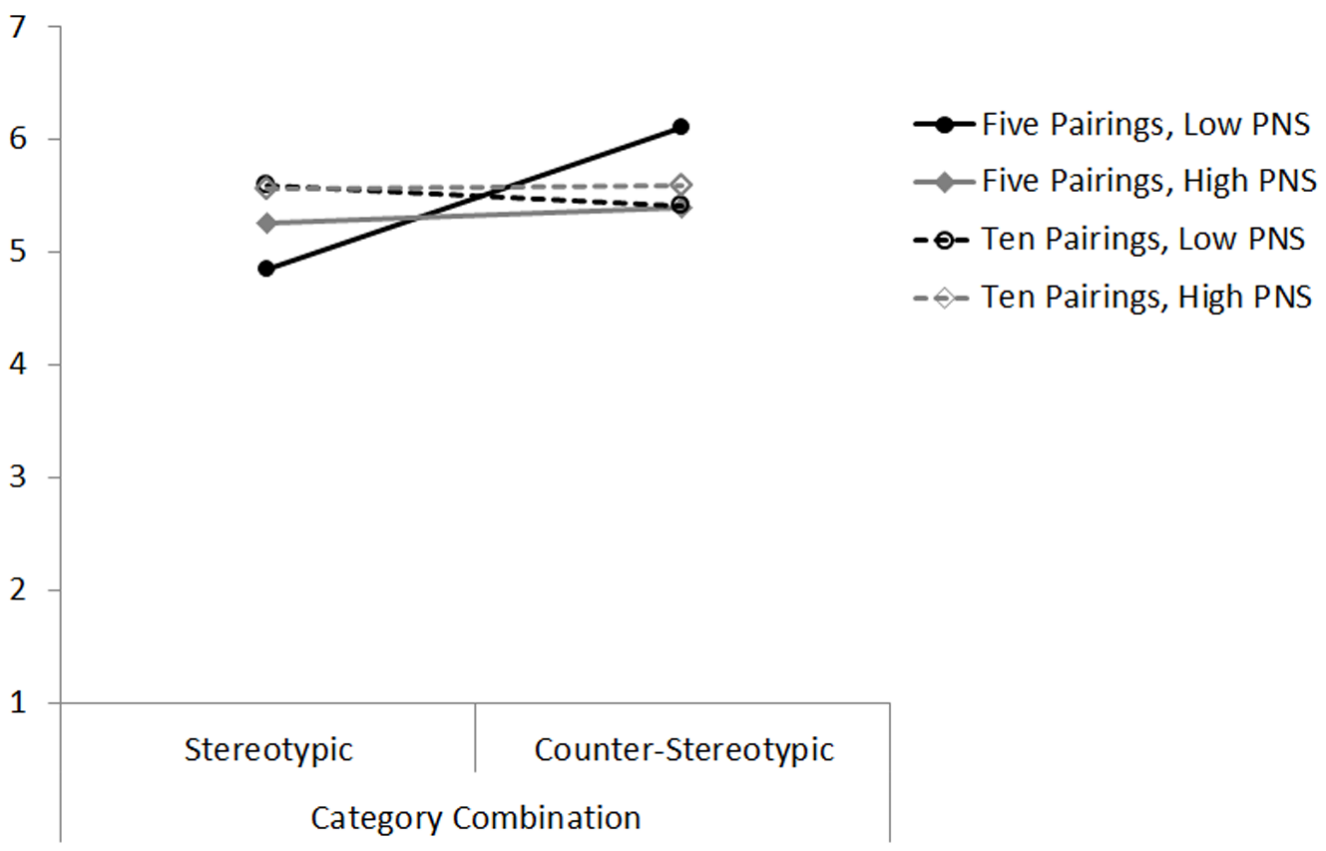
Participants’ levels of generalized Humanitarianism-Egalitarianism, plotted as a function of participants’ personal need for structure (PNS) and type of priming (high = 1 *SD* above the mean, low = 1 *SD* below the mean) [Experiment 5]. Lower scores indicate lower egalitarianism.

Given the findings from Experiment 4, we followed a hypothesis-driven approach [42], [43], and employed a planned contrast comparing the five counter-stereotypic category combinations condition (coded 1) against the other three cells of the design (coded 0). We regressed participants’ responses to the Humanitarian-Egalitarian scale on the centred PNS scores, the contrast coding, and the interaction of the two predictor variables [44]. The analysis revealed a marginally significant main effect of the condition contrast, β = .19, *p* = .080, qualified by a significant interaction with PNS, β = –.28, *p* = .036. The lower individuals’ need for personal structure, the more participants benefited from generating five counter-stereotypic category combinations in terms of an increase in their egalitarian values. As can be seen in Figure 1, thinking about five counter-stereotypic category combinations changed the egalitarian attitudes of individuals with a low (−1SD: *M*_S_ = 6.11 vs. 5.34), β = .41, *p* = .006, but not with a high need for personal structure, (+1SD: *M*_S_ = 5.39 vs. 5.44), β = –.02, *p* = .878.

We also conducted an analysis using the General Linear Model (GLM), where we added combination type and number of combinations (both categorical) as well as PNS (continuous) as predictors of Humanitarianism. Replicating Experiment 4, the results revealed an interaction between combination type and number of combinations, *F*(1, 72) = 4.79, *p* = .032, η^2^ = 0.06, which upon closer examination was driven by a significant difference between stereotypic and counter-stereotypic pairings that only emerged for five category combinations, *F*(1, 36) = 6.10, *p* = .018, η^2^ = 0.13, but did not emerge for ten category combinations, *F*<1. However, this pattern also differed depending on participants’ level of PNS. For those scoring low on PNS (−1 SD), generating five counter-stereotypic category combinations resulted in more egalitarian attitudes than generating five stereotypic category combinations, *F*(1, 36) = 8.37, *p* = .006, η^2^ = 0.17. In contrast, participants scoring high on PNS (+1 SD) were unaffected, *F*<1. This pattern did not emerge for ten category combinations, where participants’ level of PNS had no effects (*F*s<1). The overall outcome was a marginally significant three-way interaction between combination type, number of combinations, and PNS, *F*(1, 72) = 3.32, *p* = .072, η^2^ = 0.04.

These findings provide further evidence that thinking about a few surprising, counter-stereotypic social category combinations fosters a cognitive flexibility mindset that challenges established knowledge structures. Furthermore, these results show that thinking about counter-stereotypic exemplars can lead to greater endorsement of egalitarian values. Changing people’s value orientations is an important feat in the quest for generalized reduction of prejudice and greater tolerance, since values are higher order constructs that are more difficult to change and are predictive of more specific attitudes [1], [41], [45], [46].

## Experiment 6: Field Test

The aim of our final study was to test the viability of counter-stereotypic priming in the field where real conflict defines intergroup relations. The present experiment was conducted in the Former Yugoslav Republic of Macedonia and focused on attitudes towards ethnic groups that shared a history of conflict. The experiment utilized five category combinations only, which had proved most successful in the laboratory. In this study we aimed to replicate the generalized reduction of prejudice found in Experiments 4 and 5, but this time with ethnic outgroups [37]. Furthermore, we aimed to extend the findings on generalized reduction of prejudice by testing whether a counter-stereotypic mindset can also lead to greater trust and willingness to reconcile with the outgroup [47].

### Method

#### Participants and design

Eighty-four volunteering ethnic Macedonians (61 female, *M_age_ = *23.92) were randomly allocated to one of two experimental conditions (combination type: counter-stereotypic vs. stereotypic).

#### Procedure and materials

Participants were recruited from workplaces and universities in the capital of Macedonia, Skopje. Upon consenting to take part, participants were asked to generate either five counter-stereotypic, or five stereotypic category combinations. Participants then rated their attitudes toward four different ethnic outgroups: Gypsies, Albanians, Greeks, and Serbs, by using the following bipolar adjective pairs separated by a 7-point scale: *warm-cold*, *negative-positive*, *friendly-hostile*, *suspicious-trusting*, *respect-contempt*, *admiration-disgust* (*General Evaluation Scale*) [37]. Participants also rated their *generalized trust* towards the ethnic groups [47] (e.g., “Members of the ethnic minorities will exploit me if I trust them” *(R)*; 1 = *strongly disagree* to 5 = *strongly agree*) [*α* = .67, *M* = 2.62, *SD* = .88].

### Results and Discussion

A MANOVA on attitudes towards the four outgroups yielded a significant main effect, *F*(4, 78) = 6.60, *p*<.001, η^2^ = 0.09. Participants who generated counter-stereotypic category combinations displayed more positive attitudes towards the four ethnic outgroups than participants who generated stereotypic category combinations, (*Ms* = 4.05 vs. 3.72) [see Table 2]. A repeated measures ANOVA with combination type as a between-subjects factor confirmed that the effects of the counter-stereotypic priming did not differ between the four target groups, *F*(3, 243) = 1.32, *p* = .268, *ε = *.921.

**Table 2:** Attitudes towards different ethnic outgroups as a function of combination type (Experiment 6).

	Stereotypic		Counter-stereotypic	
Outgroup	*M*	*SD*	*M*	*SD*
Gypsies	3.62	.64	3.89	.70
Albanians	3.71	.58	3.87	.62
Greeks	3.36	.54	3.87	.69
Serbs	4.20	.65	4.56	.45

An Independent Samples *t*-test revealed that thinking about surprising category combinations increased trust towards the four ethnic outgroups, *t*(82) = −3.61, *p* = .001, d = 0.80 (*Ms* = 2.93 vs. 2.28). These findings demonstrate that a cognitive flexibility mindset induced by generating counter-stereotypic category combinations can succeed in reducing prejudice and fostering trust outside the laboratory in a context marked by a history of ethnic conflict.

## General Discussion

Philosophers, sociologists, politicians and policy-makers have long struggled with the problem of prejudice, with the ultimate aim of eradicating prejudice from human societies [48], [49], [50], [51], [52]. The present research adds a new psychological contribution to these efforts. We utilized principles of multiple categorization to develop a new mindset induction approach to reducing prejudice and promoting more positive intergroup relations. Across six experiments, priming a counter-stereotypic mindset increased cognitive flexibility, lowering the need for cognitive closure (Experiment 1), increasing the inhibition of dominant responses (Experiment 2), and heightening lateral thinking (Experiment 3). Most importantly, priming counter-stereotypic thinking lowered prejudice toward a multitude of outgroups (Experiments 4 and 6), increased commitment to democratic norms (Experiment 4), fostered egalitarian values (Experiment 5), and enhanced trust towards outgroups in a setting marked by a history of violent ethnic conflict (Experiment 6).

These findings underscore the potential of multiple categorization as a tool to lower prejudice, and demonstrate for the first time that multiple categorization based on surprising category combinations can induce a mindset capable of lowering generalized prejudice and increasing tolerance towards multiple outgroups. Counter-stereotypic thinking reduced Stroop interference, pointing towards increased cognitive control and the inhibition of automatic associations. Furthermore, priming counter-stereotypic thinking reduced individuals’ need for cognitive closure and improved their performance on lateral thinking tasks, suggesting an epistemic motivation to process information deeper, and in novel ways [53].

A remarkable finding is that the counter-stereotypic intervention elicited heightened trust towards multiple ethnic outgroups in a society marked by recent, visceral inter-ethnic hostilities. Intergroup trust is acknowledged to be a fundamental deciding factor whether two warring groups engage in reconciliation [54]. However, thus far research in this area has been very scant, mainly arising from the difficulties of studying real conflicted groups. From the few studies that have tested trust in intergroup conflict we know that contact predicts trust positively, thus leading researchers to propose that conflicted groups should be encouraged to come into contact more often [55]. However, one often overlooked obstacle to establishing positive contact between conflicted groups is the segregated nature of societies in conflict. Contact cannot be forced, and even when it is established it requires time for the positive benefits to occur. Therefore, there is a need for simple interventions that would make people more willing to reconcile with opponents. The present research provides evidence for increased generalized trust after generating five counter-stereotypic category combinations. Namely, thinking about the diversity that defines modern societies leads to increases in trust which in turn should lead to greater willingness to engage in positive relations. Thinking about multiple categories could provide a new, simple intervention technique to lay the grounds for increased trust and reconciliation among conflicting parties.

The fact that the novel task we used does not include a specific outgroup target may explain why it had more success than previous interventions at promoting *generalized* tolerance. The counter-stereotypic category combinations generated differed widely between participants, and more importantly these combinations differed from the multiple target outgroups that were used as a measure of generalized prejudice reduction. These characteristics of the novel task may explain the generalizability effect found in our studies. The present research has therefore shown that it is possible to affect variables that are resistant to change such as values, personal beliefs, and attitudes by changing people’s cognitive styles.

One question that arises is how the present findings can be reconciled with past research that has shown that counter-stereotypic exemplars are often assigned to a new category of unrepresentative group members? This so-called *subtyping* process enables people to maintain their pre-existing stereotypical beliefs [56], [57], [58]. Closer inspection reveals important differences between contexts that trigger subtyping and the present intervention based on counter-stereotypic category combinations. In particular, subtyping ensues in the presence of further, often neutral information (e.g., an introverted lawyer *working in a small or big firm*), but it tends not to occur when only category information is available (e.g., an introverted lawyer) [59]. This suggests that the absence of any additional person information might in fact be a critical feature of the success of the present mindset intervention. Furthermore, thinking of more than one counter-stereotypic exemplar could also counteract subtyping processes as categorical knowledge becomes increasingly difficult to reconcile with the counter-stereotypic exemplars. The fact that subtyping plagues interventions targeted at the content of people’s stereotypes (i.e., *what* people think about others) underscores the need for interventions targeted at people’s core cognition (i.e., *how* people think about others).

From a practical, applied point of view the most valuable contribution of the present research is the finding that a relatively simple, short, and inexpensive task can foster tolerance and reduce prejudice. The simplicity of the task makes it appealing and manageable to implement by practitioners in real world settings, where an ideal intervention should be quick, concise, and easy to implement. With this in mind, it is encouraging that a brief task appears to yield the most positive outcomes. The novel task can also be used in highly segregated and antagonistic settings, thus avoiding the pitfalls that previous interventions have suffered from.

A fully-fledged intervention programme may begin with the novel task, and later when participants have adapted to resolving inconsistencies arising from the counter-stereotypic challenging diversity, practitioners may introduce a contact intervention whereby people from opposing groups are brought together under optimal conditions. In this case the novel task would make people more open-minded and flexible, thereby decreasing the associated anxiety and resistance that would impede the positive effects of contact, paving the way for another more specific intervention to work. For example, someone primed with a counter-stereotypic mindset may, as a result of enhanced cognitive flexibility, engage in more contact with their former enemy, or even join superordinate category teams with outgroupers. A cognitive flexibility mindset induction could thus be the first step in a carefully designed intervention programme.

### Future Research

One important aspect that future studies should test is the long-term impact of this task. Previous research on self-regulation of prejudice has shown that the motivation to avoid expressions of negative stereotypes can lead to the automatic suppression of stereotypes over time [60], [61], [62], [63]. This ability to self-regulate stereotypes becomes easier with practice, which led Crisp and Turner [23] to suggest that repeated engagement in resolving stereotypic inconsistencies should improve the ability to suppress existing stereotypes and engage in more generative systematic thought. Importantly, as predicted by the CPAG model [23] the temporary shifts in cognitive flexibility that ensue after thinking of counter-stereotypic diversity should lead to chronic changes in people’s cognitive style of thinking. Exploring the long-term implications of chronic exposure to diversity is an important avenue for future research. On a related note, it would also be interesting for future research to explore whether asking participants to generate non-social counter-stereotypic category combinations would produce different effects to the ones obtained with the social version of the task. Another, related question pertains to the age at which interventions based on cognitive mindsets would be maximally effective. Using the task as part of personal and social education in schools may lead to benefits for future intergroup relations.

One could suggest that the present findings are driven by an increase in prejudice after asking participants to generate stereotypic social category combinations. Generation of stereotypic combinations was chosen as the most appropriate control task, keeping all other factors constant apart from the key variable of interest: stereotypicality of the generated combinations. What is more, previous research has shown that stereotypic exemplars are more easily accessible and typically guide individuals’ judgements and behavior, thus exemplifying the default thinking mode [13], [14], [15], [25], [26], [27]. Furthermore, in Experiment 3 we utilized a baseline condition in which participants did not generate any combinations prior to giving their answers on the dependent measures. The data in this experiment demonstrated that the effects on the dependent variable were driven by the counter-stereotypic condition, whereas there were no significant differences between the participants who generated stereotypic social category combinations and those participants who were in the baseline condition. These results coupled with previous theoretical and empirical findings underscore the notion that counter-stereotypic thinking can be a catalyst for improved intergroup attitudes.

The present studies also explored factors that strengthen or weaken the outcomes of the mindset induction. The CPAG model predicts that only those individuals who are motivated to engage in the inconsistency resolution process that arises when being faced with counter-stereotypically challenging diversity will show increases in cognitive flexibility. Consistent with these conjectures, Experiment 5 showed that individuals who were high in personal need for structure did not demonstrate reductions in generalized prejudice after the task. Individuals with a high personal need for structure find it harder to do away with established categories and pre-conceptions, thus providing direct evidence for the importance of categorization processes in the findings described here. Other, related individual difference constructs such as the *Need for Cognition (NFC)* [64], [65], [66], which describes an inclination for reflective thought, and *Ingroup Identification*, which denotes the degree to which individuals define or see themselves as group members [67], may have similar, moderating effects. In practical terms, these findings are important because they highlight the necessity for further research to develop tasks that succeed in inducing a counter-stereotypic mindset in people with a high personal need for structure.

As pointed out earlier in this paper, categorization is a useful tool which saves cognitive resources and time. However, the present research shows that categories may not be useful at all times, and in fact there may be discernible benefits for intergroup harmony if categories were sometimes not used at all. Therefore, it would be interesting to examine the evolutionary trajectory of categorization principles to determine the extent to which categorization into in- and outgroups is a rudimentary function from our evolutionary past, when instant categorization into different groups may have engendered benefits for survival. Evolutionary theories posit that the environment sometimes changes faster than the ability of the organism to adapt, and the result of this is that the organism finds itself mismatched to the environment. Such an effect has been postulated for rudimentary emotional responses, and may also apply to categorization processes [68]. The mismatch hypothesis has also implications for the social identification of individuals. Thus, even though identification with social groups brings a host of benefits, such as positive distinctiveness, the increasingly globalized and multicultural world we are living in may mean that intergroup differentiation is no longer a maximally viable method of perceiving the social environment.

### Conclusions

We have demonstrated that a generalized reduction of prejudice can be achieved by instigating a counter-stereotypic mindset. This work offers a possible answer to the question that has eluded social psychologists for decades, namely how to foster tolerance and reduce prejudice towards multiple outgroups. These findings have important ramifications for future theorizing in the field of prejudice reduction. The fact that the novel task does not feature a particular outgroup may explain why, in contrast to previous interventions, we observed generalized tolerance. Furthermore, by changing people’s cognitive styles the present research has shown that it is possible to affect variables that are resistant to change such as values, personal beliefs, and attitudes. The key to prejudice reduction may therefore lie in changing peoples’ *way* of thinking, rather than the content of their stereotypes.

## Supporting Information

Material S1 Cognitive task.
(DOC)
