# Supplementary material for: Tolerance by Surprise: Evidence for a Generalized Reduction in Prejudice and Increased Egalitarianism through Novel Category Combination
Source: PLoS One. 2013 Mar 6;8(3):e57106. doi: 10.1371/journal.pone.0057106 (PMC3590200; doi:10.1371/journal.pone.0057106)
Supplement: Material S1 — Cognitive task. (DOC) [file pone.0057106.s001.doc]

**Supplementary Material (S1): Cognitive task**

The first part of this study is about people’s perceptions of social groups.

The first thing we would like you to do is think generally about *social groups*. There are many different social groups or categories that people can be members of: For instance “woman”, “black”, “student”, “brother”, “footballer” …

In particular, we would like you to list below **pairings** of group membership that **tend not to go together.** In other words, pairs of group memberships that you might be *surprised to find in the same person*. Please list below five of these pairings.

| 1. |  | AND |  |
| --- | --- | --- | --- |
| 2. |  | AND |  |
| 3. |  | AND |  |
| 4. |  | AND |  |
| 5. |  | AND |  |
